# Examining the role of action-driven attention in ensemble processing

**DOI:** 10.1167/jov.24.6.5

**Published:** 2024-06-06

**Authors:** Kristina Knox, Jay Pratt, Jonathan S. Cant

**Affiliations:** 1 Department of Psychology, University of Toronto, Toronto, Canada; 2 Department of Psychology, University of Toronto Scarborough, Scarborough, Canada

**Keywords:** ensemble processing, attention, visual perception, action effect, action-driven attention

## Abstract

Ensemble processing allows the visual system to condense visual information into useful summary statistics (e.g., average size), thereby overcoming capacity limitations to visual working memory and attention. To examine the role of attention in ensemble processing, we conducted three experiments using a novel paradigm that merged the action effect (a manipulation of attention) and ensemble processing. Participants were instructed to make a simple action if the feature of a cue word corresponded to a subsequent shape. Immediately after, they were shown an ensemble display of eight ovals of varying sizes and were asked to report either the average size of all ovals or the size of a single oval from the set. In Experiments 1 and 2, participants were cued with a task-relevant feature, and in Experiment 3, participants were cued with a task-irrelevant feature. Overall, the task-relevant cues that elicited an action influenced reports of average size in the ensemble phase more than the cues that were passively viewed, whereas task-irrelevant cues did not bias the reports of average size. The results of this study suggest that attention influences ensemble processing only when it is directed toward a task-relevant feature.

## Introduction

Our environment is complex and contains far too much information for our visual system to process in detail at any one moment. One way to deal with this complexity is via ensemble processing, a perceptual mechanism that allows the visual system to circumvent capacity limitations to visual working memory and attention by condensing multiple sources of information into useful summary statistics (Cohen, DCohen, Dennett, & Kanwisher, 2016). For example, one does not need to process each individual tree to know they are looking at a forest. Ensemble perception plays a ubiquitous role in our everyday lives through the creation of statistical representations of our visual environment by means of summarizing complex and redundant information.

A key feature of ensemble perception is that summary statistics can be more accurately represented than information about any of the individual items from the set (Whitney & Yamanashi Leib, 2018). Ariely's (2001) seminal study demonstrated that the visual system creates statistical representations from sets of similar items. The results demonstrated that participants were able to identify the mean size of a group of items more accurately than the size of an individual item, thus dissociating the underlying mechanisms of ensemble processing from single-item processing. In addition to accurately identifying the mean of an ensemble, there is a common finding that single-item reports are biased toward the mean. Brady and Alvarez (2011) examined whether the ensemble statistics of a display would bias memory for individual items when observers attempted to remember the size of multiple-colored circles. They found that observers were more likely to report the size of an individual circle as larger if the average size of the other circles of the same color was large, suggesting that observers were biased by the ensemble statistics of the display when representing a single item in visual working memory. Thus ensemble representations are powerful tools that influence how we represent visual information.

The underlying cognitive mechanisms involved in ensemble processing remain a topic of investigation. Pertinent to the present study, researchers have debated whether attention is necessary for ensemble processing to occur. On one side of the debate, several studies have suggested that ensemble processing can occur in the absence of focal attention. Notably, Alvarez and Oliva (2008) demonstrated this using a multiple object tracking task followed by a judgement of either a single item or ensemble feature. Their findings demonstrated that participants could accurately report the centroid of the moving distractors (i.e., the items that were ignored) but knew little about the location details of the individual items. This result is consistent with other findings that ensemble processing can occur in the absence of focal attention (Chen, Zhuang, Wang, Ren, & Abrams, 2020; Chong & Treisman, 2005). On the other side of the debate, several studies have concluded that ensemble processing requires attention (Myczek & Simons, 2008; de Fockert & Marchant, 2008; Hochstein, Pavlovskaya, Bonneh, & Soroker, 2015; Jackson-Nielsen, Cohen, & Pitts, 2017; Baek & Chong, 2020a; Baek & Chong, 2020b). It has been argued that ensemble perception tasks are limited by the capacity of focal attention, resulting in participants using subsampling strategies to select a few items to make a judgement about the group (Myczek & Simons, 2008). Similarly, it has also been shown that directing attention toward a single member of an ensemble set—either implicitly or explicitly—can influence average feature reports (de Fockert & Marchant, 2008). More recently, Jackson-Nielsen et al., (2017) used an inattentional blindness paradigm to examine ensemble perception in the absence of attention. In their study, participants were shown displays where one row of letters was white and the remaining rows varied in color, and they were instructed to attend to only the white letters as their memory for these letters would be tested. Importantly, on critical trials they were probed on the color diversity of the entire display. The results showed that participants were unable to notice the change in color diversity on critical trials. Based on their results, Jackson-Nielsen and colleagues (2017) argue that a way to interpret the attentional dependence of ensemble processing is through a “zoom lens” metaphor, in which attention is on a continuum between focal and distributed modes rather than in binary states.

With evidence on both sides of the debate, there remains uncertainty surrounding the role that attention plays in ensemble processing. The two main experimental paradigms that have been used to examine attention in ensemble perception are the dual-task paradigm and the inattentional-blindness paradigm. In a dual-task paradigm, participants are engaged in two concurrent tasks; however, it is possible that the selected tasks are not extremely attentionally demanding, and thus there are leftover attentional resources that could be used for ensemble processing (Cohen et al., 2016). Likewise, inattentional-blindness paradigms do not motivate participants to distribute their attention across tasks and may leave room for visual working memory to be used (Jackson-Neilsen et al., 2017). More recently, attention has also been shown to impact the process of creating an ensemble representation by favoring certain items in the set. Researchers have demonstrated that weighted averaging can occur via attentional resources, such that selective attention can be preferentially allocated to certain items in the set, thus biasing the reported average (Iakovlev & Utochkin, 2021; Kanaya, Hayashi, & Whitney, 2018; Munneke, Duymaz, & Corbett, 2022). For example, Choi and Chong (2020) have examined the idea of weighted averaging via selective attention by presenting participants with a display of eight gratings of varying sizes and either a pre-cue or post-cue to indicate an item that they would have to report. The results showed that the estimated mean size was biased towards the attended size of the item, and the mean size was overestimated when attention was directed to it before the presentation of the stimuli because of the increased apparent size. Iakovlev and Utokchkin (2021) also explored the influence of attention on ensemble perception using attentional amplification, where participants were instructed to report the average orientation of triangles of different sizes. The idea was that the large triangles would be more salient, and the small triangles would be less salient, resulting in participants average reports being biased towards the orientations of the large triangles. The results revealed that participants were unable to pay attention to salient items alone and completely ignore less salient items. Similarly, Pascucci, Ruethemann, and Plomp (2021) explored the spatial weight averaging model, which describes the weights of individual items in the set that are used to compute an average. The results of their study showed that when participants were not given specific instructions about where to direct their attention, the items to the left of the center biased the reported average most, suggesting that retinal location is an important aspect to consider when investigating preferential weighting of items in ensemble processing. Together, these studies provide converging evidence that selective attention can modulate ensemble perception.

Although there is growing consensus that attention is involved in ensemble perception, the question of the precise role attention plays in this process remains an area of interest. To investigate this, in the current study, we use a different attentional paradigm altogether to modulate attention during an ensemble perception task. Specifically, we used a robust attentional manipulation to allocate attention towards specific features of an ensemble during an ensemble-perception task to allow us to determine whether attention can facilitate or bias reports of an average feature from an ensemble. Notably, there is a body of literature that provides evidence that simple actions can be used to successfully manipulate attention (Tipper, Lortie & Baylis, 1992; Pratt & Abrams, 1994), and this is generally done without intentionally altering the perceptual qualities of the stimuli and experimental task. A classic example of this is Tipper et al.' (1992) study where participants were instructed to reach for a target on a three-by-three grid. When the target appeared, a distractor would appear simultaneously at another location. The results showed longer reaction times (RT) when the distractor was within the reach trajectory, compared to when it was outside of the reach trajectory. These findings demonstrated that performing a reaching action changed how the stimuli were prioritized, such that reach-relevant distractors received higher priority than reach-irrelevant distractors. Furthermore, the type of action has also been shown to prioritize attentional selectivity towards particular feature dimensions, such as grasping prioritizing size and orientation (Bekkering & Neggers, 2002), and reaching prioritizing luminance (Wykowska, Schubö, & Hommel, 2009; see Pratt, Eric, Taylor, & Gozli, 2015 for review). Thus, in the current study, we proposed using action-driven attentional cues to manipulate attention during ensemble processing.

Research has shown that even a very simple action, such as a button press, can influence attention. This phenomenon, which is known as the action effect, was first demonstrated by Buttaccio and Hahn (2011) where participants completed a go/no-go task before a visual search task. Buttaccio and Hahn (2011) argue that a simple action preferentially allocates attention towards features of the object, and thus a simple action can influence attentional processing. In their study, participants were instructed to make an action (i.e., a button press) when a pre-cue color word matched the color of a subsequently displayed shape and did not make an action when there was a mismatch. After the go/no-go task, participants performed a visual search task where they were presented with an array of four different shapes filled with vertical lines and were asked to identify the direction of a sole tilted line. The results showed that participants were faster to respond in the visual search task on valid trials when the target (i.e., the sole tilted line) was inside of a shape that matched the acted-on color compared to when there was a mismatch. Thus, these results supported their hypothesis that acting on an object shifts the attentional weight towards the specific features of the acted-on object in subsequent visual scenes (Buttaccio & Hahn, 2011).

In line with this work, Weidler and Abrams (2014) replicated Buttaccio and Hahn's (2011) results in four experiments and extended their work to determine the depth of processing of the acted-on object that is necessary to obtain an action effect. Specifically, in their study they altered the cue such that participants were explicitly told whether they would act on a circle or not (i.e., participants did not need to make a decision about executing an action). They also examined whether making an action that had a corresponding consequence, such as shortening the length of a trial when an action was made compared to when no action was made, was necessary for the action effect to occur. Their results demonstrated that the action effect occurs when participants know they are going to respond before the object's appearance, and therefore a decision does not need to be made about whether to make an action. Furthermore, their results showed that it is not necessary for the action to have a consequence in order to demonstrate an action effect. The action effect has also been shown to have a strong effect on attentional selection in visual search (Weidler & Abrams, 2016) and on the guidance of eye movements (Wang, Sun, Sun, Weidler, & Abrams, 2017). In the current study, we used this robust action effect to determine if the allocation of attention facilitates the extraction of summary statistics when the acted-on object is congruent with the average features of the ensemble.

To address the question of how attention is involved in ensemble perception, in the current study, we created a novel paradigm that merges the typical action effect task, a manipulation of attention, with an ensemble-perception paradigm. In all three experiments, participants completed two phases in each trial: first, an action phase, and second, an ensemble-perception phase. In the action phase, the participant's task was to either make an action or passively view a shape. To make this decision, participants were first cued with a word, and if the cue word matched with a subsequently displayed shape, they would make an action. If the cue word did not match the subsequent shape, they would passively view the screen. In the ensemble-perception phase, participants were shown an ensemble display and asked to report either the average feature of the display (Experiments 1 and 3) or the feature of a randomly probed single item from the set (Experiment 2). Participants were presented with two ovals in the response display, where the target corresponded to the average size (or the correct single-item size on single-item report trials) and the distractor size was either congruent or incongruent with the cued size from the action phase (e.g., for a congruent distractor, the participant is cued with “LARGE” in the action phase, and the distracter in the ensemble-perception phase would be the larger of the two response items). We also explore how task-relevant cues (Experiment 1) and task-irrelevant cues (Experiment 3) affect the accuracy of reporting the average size. We hypothesize that if attention is involved in ensemble processing, the action effect will bias the summary statistic representation when the acted-on (attended) feature is congruent with the distractor size. For the purposes of this study, we operationalized the action effect as a significant interaction between task (i.e., making an action towards the cued feature or viewing the cued feature) and distractor congruency. In the current set of experiments, we measure both accuracy and RT, however, the accuracy results will be our main determinate of an action effect, since participants are instructed to be as accurate as possible.

## Experiment 1

Experiment 1 investigated whether attention is involved in ensemble processing by using a task-relevant, action-driven attentional cue to direct participants’ attention toward the size of the items in the ensemble. Participants completed an action phase followed by an ensemble phase in each trial. We hypothesized that if attention is involved in ensemble processing, the action effect would be present such that accuracy is lower when the acted-on size cue is congruent with the size of the distractor. For example, if participants are cued with the word “LARGE” in the action phase, then attention would be biased towards large items in the ensemble phase, which would result in an over-estimation of the perceived average size of the ensemble. Therefore, to reiterate, we predicted that participants should be less accurate in the congruent distractor condition when participants make an action (e.g., making an action towards a large rectangle in the action phase and then the distractor is the larger of the two response items in the ensemble response phase) compared to the same condition when passively viewing the rectangle (e.g., withholding an action towards a large rectangle in the viewing phase and then the distractor is the larger of the two items in the ensemble response phase). This is presumably because initially acting on an item will allow action-driven attention to facilitate the processing of some ensemble items at encoding and subsequently bias decision-making in the response phase, but passively viewing an item will not activate this action-driven attentional effect. Furthermore, we predict that a difference in accuracy for action and viewing tasks will also be present in the incongruent distractor condition (e.g., participants make an action towards a large rectangle and the distractor is the smaller of the two response items in the response phase) such that participants will be more accurate when they make an action compared to viewing the screen. This is because here participants initially act on a large rectangle and the ensemble average, that is, the target, is the larger of the two items in the response phase. However, if attention is not involved in ensemble processing, an action effect will not affect ensemble representations and there will be no or minimal differences between action and viewing conditions for congruent and incongruent distractor conditions.

### Methods

#### Participants

We recruited 26 undergraduate psychology students at the University of Toronto using SONA (16 females; 24 right-handed; *M_age_* = 19.2 years) to complete the online experiment via Pavlovia (Bridges, Pitiot, MacAskill, & Peirce, 2020) for course credit. All participants had normal or corrected-to-normal vision and no history of neurological impairments. This research was approved by the Research Ethics Board of the University of Toronto. No participants were excluded from the analysis. To determine the number of participants needed, we based our estimates for effect size and power on previous research from Sama, Srikanthan, Nestor, and Cant (2021) (the study that inspired the experimental design of our ensemble task and stimuli for all three experiments), wherein a medium effect size was reported. We used G*Power (Faul, Erdfelder, Buchner, & Lang, 2009) and assumed a medium effect size of η^2^ = 0.06 and an alpha of α = 0.05 to determine that we needed a minimum of 23 participants to achieve a power of 0.80 for all experiments.

#### Apparatus and stimuli

Data collection occurred online via Pavlovia (Bridges et al., 2020) due to the safety restrictions set in place because of the COVID-19 pandemic. Participants were told to complete the experiment using their personal computers and that they could not use a tablet or phone. All instructions and stimuli were displayed on the screen of the participants’ personal desktop computers or laptops. The experiment was programmed and the stimuli were created using the PsychoPy Builder tool (Peirce et al., 2019). The cue words (“SMALL” or “LARGE”) were written in white text and displayed on a black background in the center of the screen, and the subsequently displayed shape was a white rectangle that was either obviously small or obviously large (72 and 288 pixels, respectively), where the large rectangle was four times the size of the small rectangle. For the ensemble displays, there were eight possible ensemble sets that were presented to the participants. Four of these ensembles had a comparatively small average diameter (60 and 80 pixels) and four had a comparatively large average diameter (100 and 120 pixels). The diameters of the individual ovals varied by increments of 10 pixels (Sama et al., 2021). The sizes of the individual ovals ranged from 25 to 115 pixels in the comparatively small displays or from 80 to 155 pixels in the comparatively large displays. The items in the ensembles maintained a minimum distance of 5 pixels from the average, and followed a uniform distribution around the mean. For example, an ensemble with a comparatively small average size of 60 pixels contained eight ovals of the following sizes: 25, 35, 45, 55, 65, 75, 85, 95. An ensemble with a comparatively large average size of 100 pixels contained eight ovals of the following sizes: 85, 95, 105, 115 125, 135, 145, 155. The ensemble was displayed for 300 ms. It is also important to note that, in addition to the stimuli in the action and ensemble phases being distinctly different in shape, the sizes of the rectangles did not overlap with the average size of the circles, and no individual oval was the same size as the average size of the entire display. The ovals in the ensemble displays were randomly presented across 12 fixed locations where six locations formed an outer ring and six locations formed an inner ring. Within each ring, four locations were filled by an oval, and two were always left empty. Because of the nature of online studies, we were unable to have full control over participants’ screen size, so we used normalized units in PsychoPy to scale the stimuli to the size of each participant's screen. We based the sizing of the stimuli on a screen with a 1024 × 768 resolution, 60-hertz refresh rate, and estimated that participants could view the screen from 50 cm away (participants were instructed to sit at approximately arm's length distance from their computer screen). We then converted the units from pixels to visual degrees and divided the degrees by 10 to convert the units into the normalized units in PsychoPy. The locations of the ensemble items were set using normalized units in PsychoPy; thus the locations were automatically adjusted based on the participant's computer screen size and the ensemble set covered approximately 78% of the participant's screen.

#### Design and procedure

This experiment used a 2 (task: action or viewing) × 2 (distractor congruency: congruent or incongruent) within-subjects design, yielding four unique conditions. The order of the conditions was randomized and counterbalanced such that each unique condition appeared 16 times within each block of trials. Participants completed 20 practice trials before beginning the experimental trials. There were 64 trials per block and six blocks in total, with each participant completing a total of 384 experimental trials.

**Figure 1.**
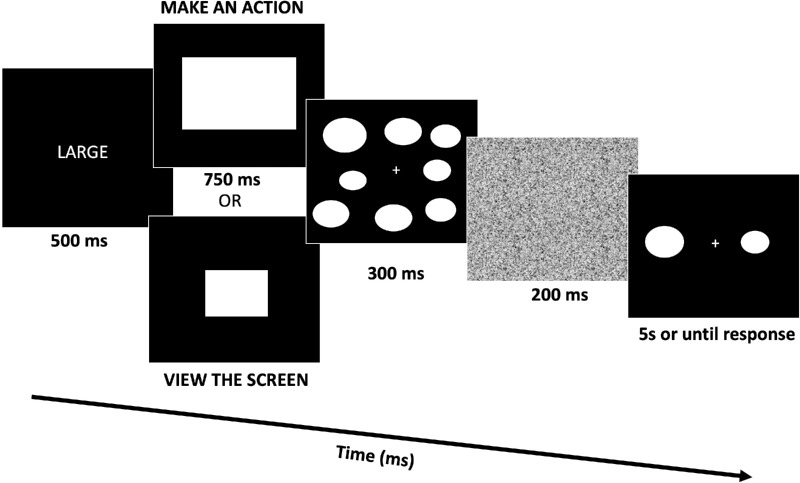
In Experiment 1, participants are shown a cue word (e.g., “LARGE”), and if the cue word matches the size of the subsequently shown rectangle, participants make an action. If the cue word does not match the size of the rectangle, participants passively view the screen. After, they are shown an ensemble display of eight ovals and are then asked to report the average size of the entire display. Note that the stimuli are not drawn to scale and are approximations.

At the start of the experiment, the instructions were displayed on the screen, and participants were instructed to place their left hand on the spacebar and their right hand on the arrow keys of the keyboard and always to maintain central fixation. Additionally, participants were instructed to view their screen at their arm's length distance (approximately 50 cm away from the screen). Participants had two main phases in each trial. The action phase was always completed first, wherein a cue word (either “SMALL” or “LARGE”) in white text was displayed on the screen for 500 ms. Next, either a small or large white rectangle was displayed on the screen for 750 ms. The Participants’ task was to press the spacebar if the cue word corresponded to the subsequently displayed object. For example, if the cue word was “LARGE” and the rectangle was also large, participants would make an action by pressing the spacebar (see Figure 1). If the cue word and object did not match, participants would not make an action and instead passively viewed the screen. Immediately after the action phase, participants began the ensemble phase, where an ensemble of eight white ovals was displayed on the screen around a central fixation cross for 300 ms, after which a 200 ms visual noise mask was presented to halt any further processing of the ensemble. After the mask, participants were given a two-alternative forced choice (2AFC) average report task. Participants were instructed to use the left and right arrow keys to indicate which of two ovals presented on the screen (to the left and right of the central fixation cross) corresponded to the mean size of the ensemble, where the size of the target oval corresponded to the mean size of the ensemble and the size of the distractor oval was always outside of the range of the ensemble (i.e., 2 standard deviations outside of the range of individual oval sizes). The distractor oval could either be congruent (e.g., a large distractor after a large-rectangle cue) or incongruent (e.g., a large distractor after a small-rectangle cue) with the cue in the action phase. We defined distractor congruency based on the size of the rectangle and the size of the distractor. It is also important to note that the distractor size was counterbalanced so that it would be larger or smaller than the target equally as often for all eight ensembles, regardless of whether the ensemble had a large or small average size. The two ovals were presented on the screen for 5000 ms or until a response was made in the 2AFC average-report task, and the spatial locations of the target and distractor were counterbalanced so that the target appeared equally often on the right and left of fixation.

#### Transparency and openness

We report how we determined our sample size, data exclusions, manipulations, and measures used in all experiments. All data, analysis code, and research materials are available on the Open Science Framework at https://osf.io/zv6na/. Data for all three experiments were analyzed using JASP version 0.16 (JASP Team, 2022) and plots were created using R version 4.0.0 (R Core Team, 2022) and the package ggplot, version 3.2.1 (Wickham, 2016). This study's design and its analysis were not preregistered.

### Data analysis

A 2 (task: action or viewing) × 2 (distractor congruency: congruent or incongruent) repeated-measures analysis of variance (ANOVA) was conducted on accuracy and RT. It is important to note that accuracy is our main variable of interest since this was not a speeded task; participants were simply instructed to respond as accurately as possible. Prior to the statistical analysis, an RT outlier analysis was conducted where RTs greater or less than 2.5 standard deviations from the mean RT for the average-report task were removed. Only trials where participants correctly made an action or correctly withheld from making an action were analyzed. Participants' accuracy in the action phase was high (*M =* 86%), and no participants were excluded from this analysis. Post-hoc *t*-test comparisons were done to analyze significant interactions using the Bonferroni correction. Additionally, we conducted a Bayesian ANOVA using the matched model method with the default priors to gauge evidence in favor of the null hypothesis (using JASP version 0.16), where a Bayes factor (BF) value of 3 or higher indicates substantial support for the null, a value between 1 and 3 indicates weak support for the null, and a value of 1 or lower indicates support for the alternative hypothesis (Jeffreys, 1961). Finally, we also conducted an exploratory analysis to explore how different definitions of congruency and incongruency affect our results (i.e., defining congruency as the relationship between the word cue and the distractor size).

### Results

**Figure 2.**
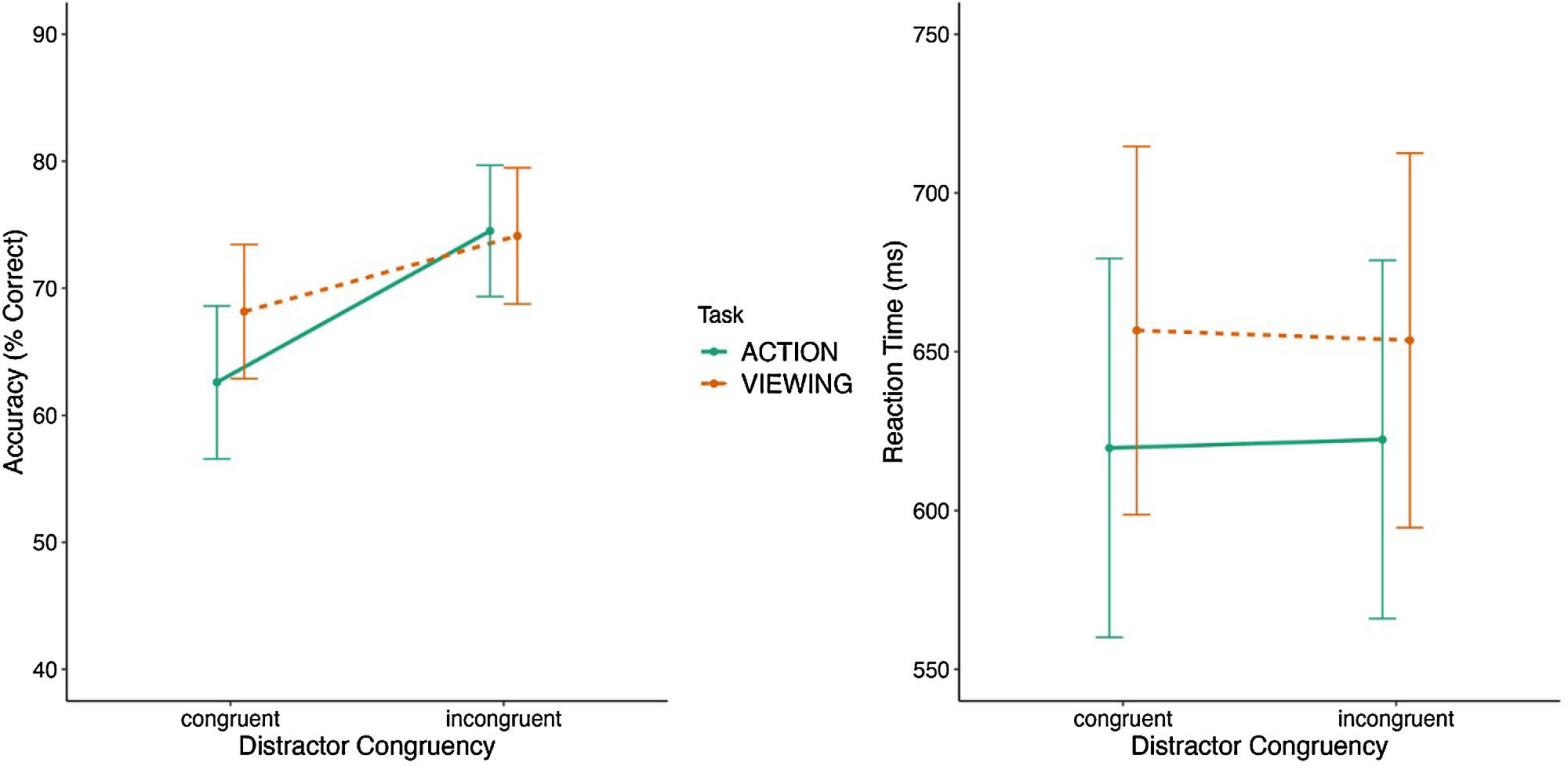
Results of Experiment 1. The left panel shows the accuracy results of the ensemble phase for congruent and incongruent displays, and the right panel shows the RT results of the ensemble phase for the same conditions. The error bars in both graphs represent the 95% confidence intervals.

The analysis on accuracy in the ensemble phase revealed a significant main effect of task [*F*(1, 25) = 8.493, *p* < 0.01, η^2^ = 0.032, BF_excl_ = 0.867], with lower accuracy for the action task (*M* = 69%, *SE* = 2%) compared to the viewing task (*M* = 71%, *SE* = 2%) (see Figure 2). There was also a significant main effect of distractor congruency [*F*(1, 25) = 29.102, *p* < 0.001, η^2^ = 0.379, BF_excl_ = 1.553 × 10^−7^], with lower accuracy for the congruent condition (*M* = 65%, *SE* = 2%) compared to the incongruent condition (*M* = 74%, *SE* = 2%). Importantly, there was a significant two-way interaction between task and distractor congruency [*F*(1, 25) = 8.217, *p* < 0.01, η^2^ = 0.042, BF_excl_ = 0.340].

Based on our a priori predictions, we investigated the two-way interaction in greater detail. Specifically, we examined differences in accuracy between the action and viewing tasks separately for the congruent and incongruent distractor conditions. For the congruent distractor condition, participants were significantly less accurate in the action task compared with the viewing task [t(25) = 4.073, *p_bonf_* = 0.001, *d* = −0.744, BF_01_ = 0.025], but the difference between the action and viewing tasks was not significant for the incongruent distractor condition [t(25) = 0.286, *p_bonf_* = 1, *d* = 0.061, BF_01_ = 4.616].

The analysis on RT revealed a significant main effect of task [*F*(1, 25) = 13.234, *p* = 0.001, η^2^ = 0.215, BF_excl_ = 0.001], with faster RT for the action task (*M* = 620 ms, *SE* = 29 ms) compared to the viewing task (*M* = 654 ms, *SE* = 29 ms). There was no significant main effect of distractor congruency [*F*(1, 25) = 0.977, *p* = 0.565, η^2^ = 8.694 × 10^−^^6^, BF_excl_ = 4.925], because RTs were similar in the congruent (*M* = 637ms, *SE* = 4ms) and incongruent displays (*M* = 637ms, *SE* = 5 ms). The two-way interaction between task and display congruency was also not significant [*F*(1, 25) = 0.341, *p* = 0.565, η^2^ = 0.002, BF_excl_ = 3.253] (see Figure 2).

The exploratory analysis in which we defined congruency based on the word cue and the size of the distractor oval revealed similar results to our original analysis, such that there was a significant main effect of task [*F*(1, 25) = 8.493, *p* < 0.05, η^2^ = 0.032, BF_excl_ = 1.606], with lower accuracy for the action task (*M* = 68%, *SE* = 2%) compared to the viewing task (*M* = 71%, *SE* = 2%). There was also a significant main effect of distractor congruency [*F*(1, 25) = 8.217, *p* < 0.05, η^2^ = 0.42, BF_excl_ = 1.098], with lower accuracy for the congruent condition (*M* = 65%, *SE* = 2%) compared to the incongruent condition (*M* = 74%, *SE* = 2%). Importantly, there was a significant two-way interaction between task and distractor congruency [*F*(1, 25) = 29.102, *p* < 0.001, η^2^ = 0.379, BF_excl_ = 5.208 × 10^−^^8^].

### Discussion

The accuracy results of Experiment 1 demonstrate an action effect, such that the significant interaction between task and distractor congruency revealed that participants were significantly less accurate in the congruent distractor condition when reporting average size after making an action compared to passive viewing. It is also worth noting that the presence of the action effect was not dependent on the way congruency between the cue and distractor was defined. We predicted that the difference would occur because of an over- or underestimation of the average size corresponding to the task-relevant cue. Unexpectedly, this pattern was not observed in the incongruent distractor condition, in which there was no difference in accuracy between the action and viewing tasks. It's possible that no difference was found in the incongruent condition because the distractor size no longer matched the over- or underestimated average size representation. Additionally, the RT results showed that participants did not trade off accuracy for more quickly initiated responses, because participants were not instructed to make a speeded response. Overall, the accuracy results provide evidence that action-driven attention influences the processing of average size from ensemble displays.

## Experiment 2

It is known that there is a distinction between ensemble processing and single-item processing (Whitney & Yamanashi Leib, 2018). Specifically, when viewing ensemble displays, participants are able to report summary statistics more accurately than features of single items (Ariely, 2001). To investigate whether action-driven attention would bias single-item processing within the context of an ensemble, we used the action-effect task to manipulate attention and asked participants to report the size of a single item from a previously seen ensemble. If attention is involved in processing and recalling features from individual items within an ensemble, the action effect should bias the encoding of an individual item when the acted-on size is congruent with the size of the distractor. This would result in a difference in accuracy between the action and viewing tasks. However, if attention plays no role in this process, then the action effect will not facilitate performance when reporting the size of single items, such that there will be no difference in accuracy between the action and viewing tasks for the congruent and incongruent distractor conditions when participants make an action.

### Methods

#### Participants

We recruited 25 undergraduate psychology students at the University of Toronto (14 females; 24 right-handed; *M_age_* = 19.2 years) as participants using SONA to complete the online experiment via Pavlovia for course credit. All participants had normal or corrected-to-normal vision and no history of neurological impairments. This research was approved by the Research Ethics Board of the University of Toronto. No participants were excluded from the analysis.

#### Apparatus and stimuli

The apparatus and stimuli used in Experiment 2 were identical to Experiment 1.

#### Design and procedure

Experiment 2 followed the same design and procedure as Experiment 1, with the only change being that participants reported single size, rather than average size, in the 2AFC component of the ensemble phase. As in the previous experiment, each trial began with the action task. Immediately after the action task, participants completed the ensemble task. In this experiment, the spatial location of the oval to be reported was cued with an arrow on the response screen, and participants were instructed to report the size of a single item from the previously seen ensemble set.

### Data analysis

The analysis for Experiment 2 is identical to Experiment 1. Participants’ accuracy in the action phase was high (*M =* 86%), and no participants were excluded from this analysis.

### Results

**Figure 3.**
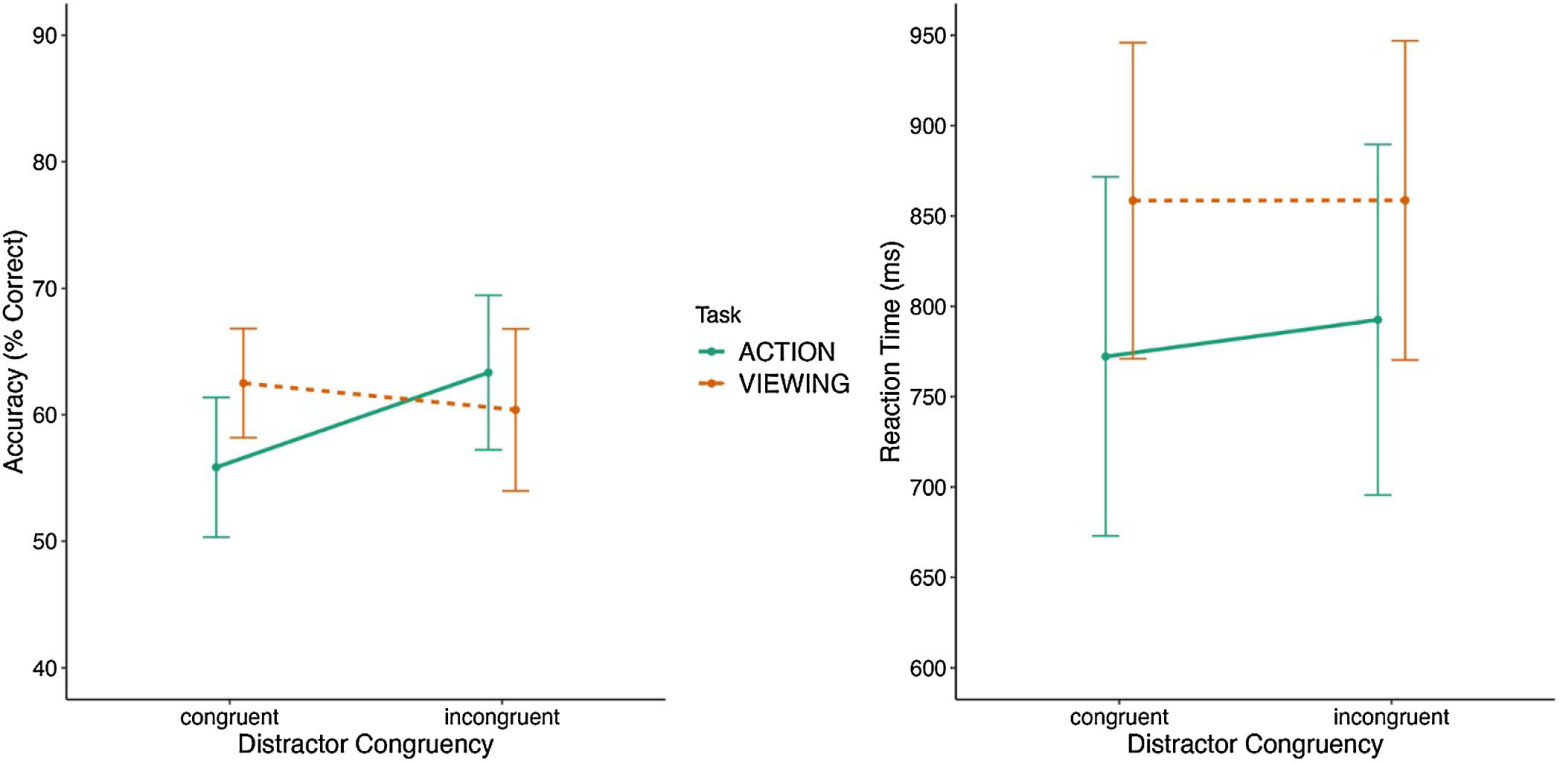
Results of Experiment 2. The left panel shows the accuracy results of the ensemble phase for congruent and incongruent displays, and the right panel shows the RT results of the ensemble phase for the same conditions. The error bars in both graphs represent the 95% confidence intervals.

The analysis on accuracy revealed a significant main effect of task [*F*(1, 24) = 4.512, *p* < 0.05, η^2^ = 0.009, BF_excl_ = 3.447], with lower accuracy for the action task (*M* = 59%, *SE* = 2%) compared to the viewing task (*M* = 61%, *SE* = 2%; see Figure 3). There was no significant main effect of distractor congruency [*F*(1, 24) = 2.737, *p* = 0.111, η^2^ = 0.020, BF_excl_ = 2.490], and the critical two-way interaction between task and distractor congruency was also not significant [*F*(1, 24) = 2.213, *p* = 0.150, η^2^ = 0.063, BF_excl_ = 0.466].

The analysis on RT revealed a significant main effect of task [*F*(1, 24) = 15.28, *p* < 0.001, η^2^ = 0.309, BF_excl_ = 1.759 × 10^−5^], with faster RT for the action (*M* = 782 ms, *SE* = 29 ms) compared to the viewing task (*M* = 858 ms, *SE* = 29 ms; see Figure 3). Similar to the accuracy results, the main effect of distractor congruency was not significant [*F*(1, 24) = 1.064, *p* = 0.313, η^2^ = 0.006, BF_excl_ = 3.701], and the two-way interaction between task and distractor congruency was also not significant [*F*(1, 24) = 1.906, *p* = 0.180, η^2^ = 0.005, BF_excl_ = 2.780].

### Discussion

An action effect was not present in the single-size report task. Although the accuracy findings demonstrate a main effect of task, there is no effect of the distractor congruency with the cue, and importantly, no interaction between the task and distractor congruency factors. Similarly, the RTs results, although not our main variable of interest, also show a main effect of task, but no main effect of distractor congruency or an interaction between task and distractor congruency. Additionally, the RT results showed that participants did not trade-off accuracy for more quickly initiated responses, since participants were not instructed to make a speeded response. Thus the findings reveal that making an action toward a congruent cue does not incorrectly bias the reporting of the size of a single item recalled from a previously seen ensemble. Therefore we can conclude that modulating action-driven attention does not bias the processing of single items in an ensemble display, despite such an effect being observed for the computation of the average size of the same display (see Experiment 1). Overall, the accuracy results of Experiments 1 and 2 suggest that attention is involved in ensemble processing but not in the processing of the features of a single item within an ensemble. These differences are consistent with the notion that the processing of ensemble and single-item features are mediated by separate cognitive mechanisms (Ariely, 2001; Haberman & Whitney, 2009; Cant, Sun, & Xu, 2015). However, the results of the Bayesian ANOVA on accuracy did not provide consistent evidence in favor of the null hypothesis, suggesting that further work may be needed to explore the impact of action-driven attention on single-item processing.

## Experiment 3

In Experiment 3, we examined whether the action-driven attentional cue must be relevant to the type of feature that is being extracted from the ensemble displays. Specifically, it is unclear if the action effect holds in situations where the visual features used in the action and ensemble phases are uncorrelated. That is, in both Experiments 1 and 2, the action-driven attentional cues we used were task-relevant (i.e., cueing the same feature being reported in the average-report task). However, there is some evidence suggesting that task-irrelevant features can also bias ensemble processing (Brady & Alvarez, 2011; Williams, Pratt, Ferber, & Cant, 2021). In this experiment, participants were first cued with a task-irrelevant feature, color, and were then asked to report the average size of an ensemble display consisting of blue and yellow ovals. In the 2AFC average-report task, participants chose between the target, the average of the entire display, and an incorrect distractor, which was the average of the subset of either the blue or yellow ovals. Including a distractor size within the range of the ensemble increased the level of interference occurring in the ensemble phase, and we expected the accuracy to be at chance (Sama et al., 2021). Thus we chose to calculate a new dependent measure, namely, the average bias for trials where participants did not choose the target (i.e., made an incorrect response). With this new measure, we operationalized the action effect as a significantly higher bias in the action condition compared to the viewing condition (see “Methods” for a description of how we calculated bias). If acting on a task-relevant feature is necessary for attention to modulate ensemble processing, then participants’ reports of the average size of the entire display should not be biased toward the average size of the subset of ovals in the display that contain the acted-on color, and an action effect will not be present. However, if acting on a task-relevant feature is not necessary for attention to modulate ensemble processing, participants’ reports of the average size of the entire ensemble will be biased by the average size of the subset of ovals sharing the same color as the cue in the action phase, and an action effect will be present.

### Methods

#### Participants

We recruited 29 participants to complete this online experiment, which again was conducted using Pavlovia (Bridges et al., 2020). Fourteen of these participants were undergraduate psychology students at the University of Toronto and were recruited using SONA and compensated with course credit, and 15 were recruited from Prolific (2023) and compensated with payment (13.57 CAD/hour). Because there were no significant differences in performance between the participant pools, the separate data sets were combined for the full analysis reported below. Four participants were excluded because of their accuracy in the action phase being less than three standard deviations from the grand mean for accuracy across all participants. Thus the final sample size used in the analysis was 25 participants (16 females; 21 right-handed; *M_age_* = 22.2 years). All participants had normal or corrected-to-normal vision, normal color vision, and no history of neurological impairments. Prolific participants were geographically restricted to North America, Ireland, and England, and were required to be fluent in English. This research was approved by the Research Ethics Board of the University of Toronto.

#### Apparatus and stimuli

**Figure 4.**
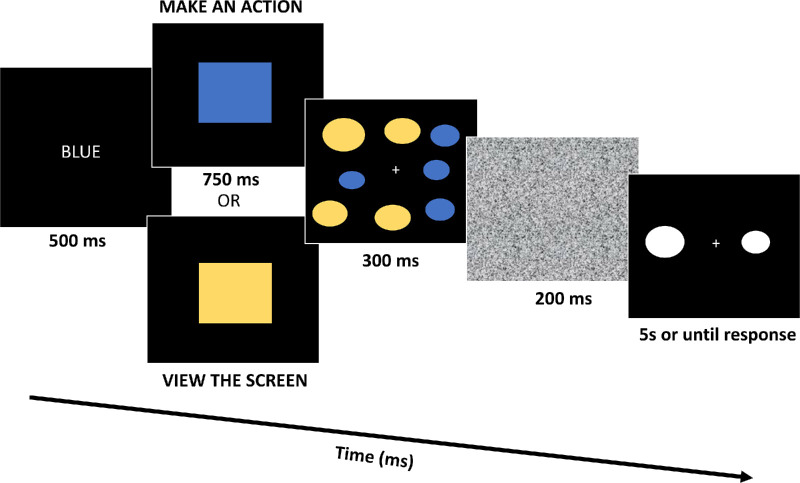
In Experiment 3, participants are shown a cue word (e.g., “BLUE”), and if the cue word matches the color of the subsequently shown rectangle, participants make an action. If the cue word does not match the color of the rectangle, participants passively view the screen. Next, they are shown an ensemble display of eight ovals and then asked to report the average size of the entire display. Participants are shown the global average of the ensemble (the target) and one of the color group (yellow or blue ovals) averages as the distractor in the 2AFC average-report task. Note that the stimuli are not drawn to scale and are approximations.

Experiment 3 used the same apparatus and stimuli as Experiments 1 and 2, with the only changes being the cue words and the colors of the cued rectangles and the ovals in the ensemble display (see Figure 4). Specifically, participants were cued with the word “BLUE” or “YELLOW”, and blue or yellow rectangles of the same size were presented after the cue word, and for each ensemble display, half of the ovals were colored blue, and half were colored yellow.

#### Design and procedure

The procedure is identical to Experiment 2 except for the following changes. In the action phase participants were cued with color words (either “YELLOW” or “BLUE”) and then shown colored rectangles of the same size that either promoted or inhibited an action, and in the ensemble phase they were shown an ensemble of eight ovals consisting of four yellow and four blue randomly sized ovals and were asked to report the average size of the entire ensemble display (i.e., the average of all eight yellow and blue ovals). On the response screen, one oval was a correct target, which was the average size of the entire display, and one oval was an incorrect distractor, which was the average of the subset of blue ovals on 50% of the trials and the average of the subset of the yellow ovals on 50% of the trials. It is important to note that the size of the distractor oval was never equal to the size of the target oval; however, the size of the distractor was within the ensemble's range of sizes, making the likelihood of interference from the distractor high (i.e., when the feature value of a distractor is within the range of an ensemble, it interferes with the representation of the ensemble average and affects performance by decreasing overall accuracy; Sama et al., 2021).

### Data analysis

Our main analysis consisted of conducting two-tailed repeated measures *t*-tests to compare percent average bias and RT in the action task compared to the viewing task. Before the analysis, we conducted an RT outlier analysis in which trials where RTs greater or less than 2.5 standard deviations from the grand mean RT were removed. Only trials where participants correctly made an action or correctly withheld from making an action were analyzed. After excluding four participants, the accuracy for the action phase was high (*M* = 87%). As stated previously, we expected the accuracy for the ensemble phase to be close to chance with the inclusion of a high interference distractor (i.e., a distractor that was within the range of size values of the previously seen ensemble; Sama et al., 2021). Thus, although we still report accuracy (see below), as we did in Experiments 1 and 2, in Experiment 3 we focus more attention on a new dependent measure, percent average bias, which allows us to examine whether attention to task-irrelevant features modulates reports of average size. To do this, the analysis of the ensemble phase data was restricted to the trials where participants chose the distractor to examine any bias evident in choosing the average of either the blue or yellow ovals. A response was considered biased if the participant chose the average that corresponded to the color of the ovals that they were cued with in the action task (e.g., if the cue color was blue in the action phase, and they chose the average of the blue ovals in the ensemble phase). A score significantly above 50% indicates bias towards the acted-on color, a score significantly below 50% indicates bias towards the opposite color, and a score no different from 50% indicates no bias. Additionally, we explored null results using a Bayesian paired samples *t*-test with the default priors on JASP version 0.16.

Additionally, we conducted the same analysis as Experiment 1, specifically a 2 (task: action or viewing) × 2 (distractor congruency: congruent or incongruent) repeated-measures ANOVA on the correct trials for both the accuracy and RT data. Similar to Experiment 1, a congruent distractor condition occurred when the distractor size matched the average size of the acted-on color subset, and an incongruent distractor condition occurred when the distractor size did not match the average size of the acted-on color subset.

### Results

**Figure 5.**
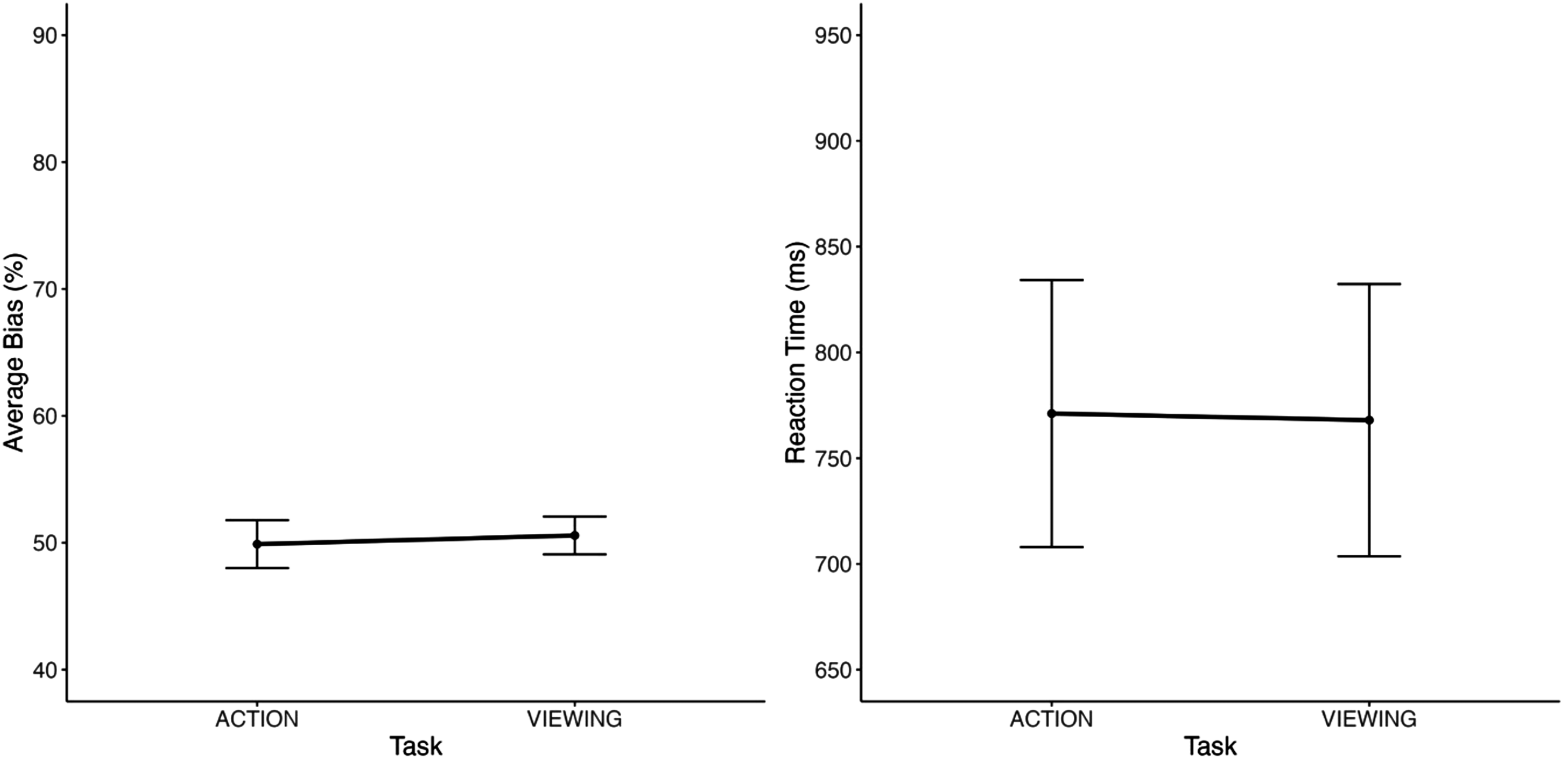
Results of Experiment 3. The left panel shows the average bias results for the action and viewing conditions, and the right panel shows the RT results for the same conditions. The error bars in both graphs represent the 95% confidence intervals.

As expected, based on the nature of a high-interference ensemble task, the participants’ overall accuracy was 50% (Sama et al., 2021). The *t*-test on percent average bias scores revealed that there was no significant difference between the percent biases observed in the action (*M* = 50%, *SE* = 0.7%) and viewing (*M* = 49%, *SE* = 0.8%) tasks [t(24) = −0.681, *p* = 0.502, *d* = −0.136, BF_01_ = 3.840] (see Figure 5). Similarly, the analysis on RT revealed no significant difference between the action (*M* = 771 ms, *SE* = 44 ms) and viewing (*M* = 768 ms, *SE* = 45ms) tasks [t(24) = 0.242, *p* = 0.811, *d* = 0.048, BF_01_ = 4.617]. Additionally, we compared the average bias scores for the action and viewing tasks to chance performance (i.e., 50%). The results revealed nonsignificant effects for the action [t(24) = −0.014, *p* = 0.989, *d* = −0.003] and viewing [t(24) = 0.787, *p* = 0.439, *d* = 0.157] tasks, indicating that performance in both conditions did not differ from chance.

**Figure 6.**
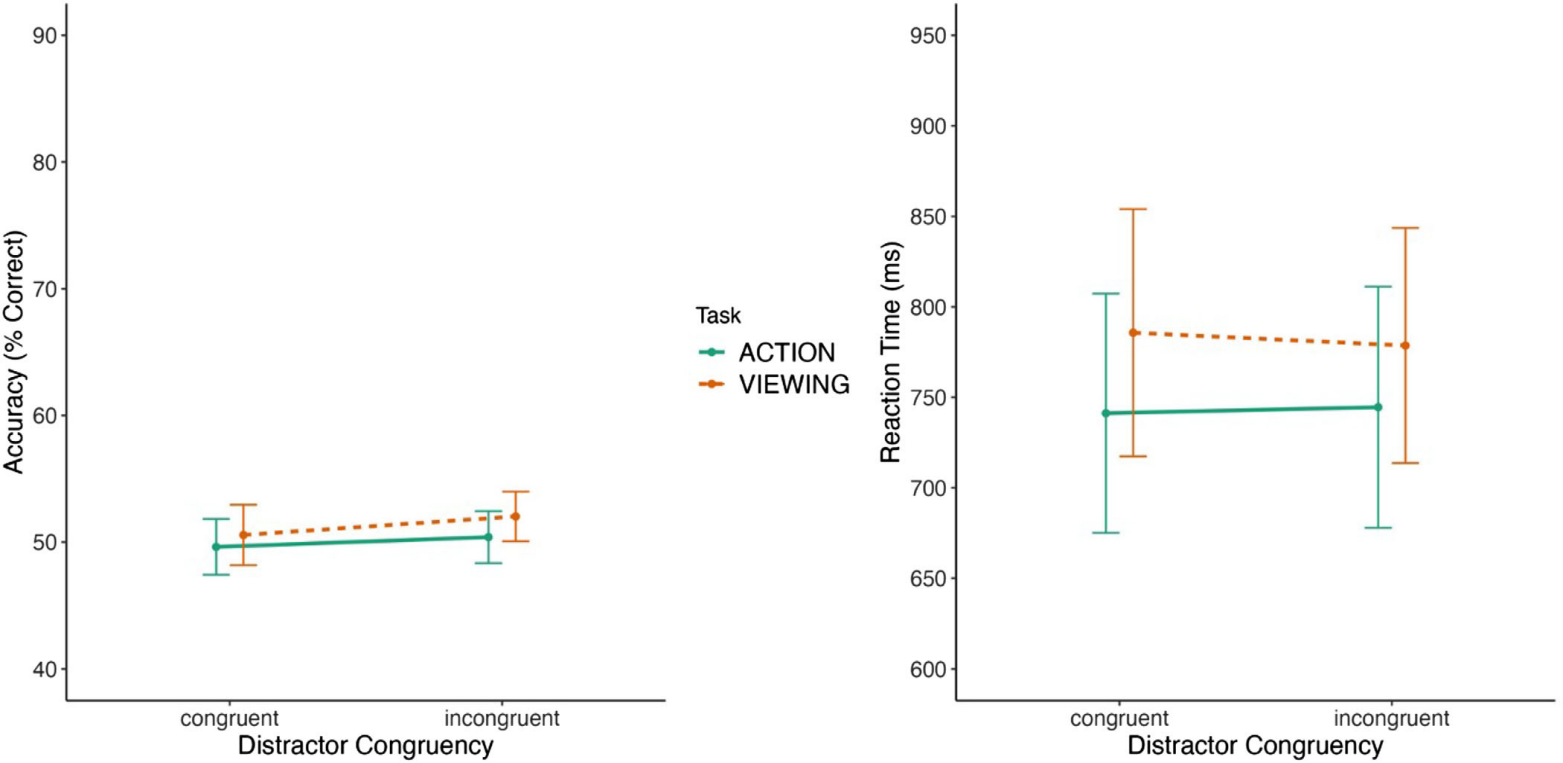
Results of Experiment 3. The left panel shows the accuracy results of the ensemble phase for congruent and incongruent displays, and the right panel shows the RT results of the ensemble phase for the same conditions. The error bars in both graphs represent the 95% confidence intervals.

The ANOVA on the accuracy data did not reveal a significant main effect of task [*F*(1, 24) = 1.723, *p* =.202, η^2^ = 0.019, BF_excl_ = 2.346], with similar accuracy for the action task (*M* = 50%, *SE* = 0.2%) compared to the viewing task (*M* = 51%, *SE* = 0.2%; see Figure 6). There was no significant main effect of distractor congruency [*F*(1,24) = 0.873, *p* = 0.359, η^2^ = 0.016, BF_excl_ = 2.703], with similar accuracy for congruent (*M* = 50%, *SE* = 0.1%) and incongruent (*M* = 51%, *SE* = 0.1%) distractor conditions. Finally, the critical two-way interaction between task and distractor congruency was also not significant [*F*(1, 24) = 0.055, *p* = 0.816, η^2^ = 0.0006, BF_excl_ = 3.465]. The analysis on RT revealed a significant main effect of task [*F*(1, 24) = 8.808, *p* < 0.05, η^2^ = 0.163, BF_excl_ = 0.013], with shorter RT for the action task (*M* = 742 ms, *SE* = 5 ms) compared to the viewing task (*M* = 782 ms, *SE* = 5 ms; see Figure 6). There was a significant main effect of distractor congruency [*F*(1, 24) = 0.103, *p* < 0.05, η^2^ = 0.0002, BF_excl_ = 4.763], with longer RTs for the congruent distractor (*M* = 763 ms, *SE* = 0.2 ms) compared to the incongruent distractor (*M* = 761 ms, *SE* = 0.2 ms). The critical two-way interaction between task and distractor congruency was not significant [*F*(1, 24) = 0.292, *p* = 0.594, η^2^ = 0.003, BF_excl_ = 3.192].

### Discussion

We did not observe an action effect in the results of Experiment 3 for either the analysis of percent bias or correct trials. Together with the finding that performance in the action task was not significantly different from chance, we conclude that directing action-driven attention towards a task-irrelevant feature does not bias ensemble perception. Additionally, we speculate that the accuracy analysis on the correct trials did not reveal an action effect because of the overall lower accuracy in the ensemble phase, due to the high interference distractor. Furthermore, we replicated the high-interference effect observed in Sama et al. (2021), wherein the presence of a distractor that is within the range of an ensemble interferes with average reports and decreases overall performance accordingly. Although it was not our main variable of interest, because participants were not instructed to make speeded responses, the RT analysis of correct trials also did not show an interaction between task and congruency. Similarly, the RT analysis of percent bias did not show a difference between action and viewing tasks, further suggesting that acting on a task-irrelevant feature (i.e., acting on a color, and then computing the average size of an ensemble display) does not influence ensemble processing (see “General Discussion” for further discussion).

## General discussion

The current study aimed to determine the role that attention may play in ensemble processing. In this series of experiments, we used the robust action effect to modulate action-driven attention towards a feature that was either task-relevant or task-irrelevant to the subsequent ensemble task. In both cases, the feature participants were cued with could either be congruent or incongruent with the distractor. Experiments 1 and 2 examined task-relevant cues, where participants were cued with size and reported the average size of the ensemble (Experiment 1) or the size of a single item in the ensemble (Experiment 2). In contrast, Experiment 3 examined task-irrelevant cues, where participants were cued with color and reported average size. Across three experiments, the results demonstrated that action-driven attention can influence ensemble processing under certain conditions. Specifically, attention influences ensemble processing when it is directed toward a task-relevant feature. In contrast, action-driven attention did not similarly affect the perception of single items within the ensemble, providing further evidence that these two processes are mediated by distinct cognitive mechanisms.

Overall, the results of this study show that attention can be involved in ensemble perception. There are, however, certain conditions under which action-driven attention can facilitate the extraction of summary statistics. Previous work exploring the role of attention focused on the mode of attentional deployment, such as focal or distributed attention (e.g., Chong & Triesman, 2005), or whether it is necessary to pay attention directly to the ensemble to accurately extract summary statistics (e.g., Alvarez & Oliva, 2008; Jackson-Nielsen et al., 2017; Chen et al., 2020). However, it is possible that attention is a tool used to influence how the ensemble is processed rather than being necessary for the process itself. For example, Cant and colleagues (2015) used the Garner speeded-classification task (Garner, 1974) to assess independence in the processing of shape and texture in ensembles and single objects, and manipulated the attentional strategy observers used to process the ensemble. Notably, they found that when ensembles were processed using a global-processing strategy, observers could not ignore changes in shape when attending to ensemble texture, and vice versa. However, processing ensemble features using a local-processing strategy (akin to how single objects are individuated from the ensemble) eliminated the interference effects. The authors suggest that these global and local processing strategies are related to attentional mechanisms, which provide the conditions for attention to influence ensemble processing. Thus we speculate that attention may be modulating the processing of ensemble features at a global level in our paradigm.

Additionally, the results of Experiment 2 did not demonstrate an action effect, providing evidence that action-driven attention does not facilitate single-item processing of items within an ensemble. While this result was surprising with regard to the action effect literature, a common finding in the ensemble perception literature is a dissociation in performance across ensemble and single-item tasks (i.e., participants are able to extract summary statistics more accurately compared to features of a single item within the ensemble; Ariely, 2001; Whitney & Yamanashi Leib, 2018). Furthermore, there is a distinction between ensemble processing and single-item processing, such that single-item recognition is not necessary for ensemble processing (Whitney & Yamanashi Leib, 2018). That is, it is not necessary for participants to encode and process single items of the ensemble in detail when forming a summary statistic representation from the ensemble. Given this, it is likely that the lack of bias of action-driven attention for single-item processing within the context of an ensemble is due to differences in how attention modulates the processing of ensembles versus single items. Thus, the results of Experiment 2 are consistent with the finding that ensemble processing and single-item processing are mediated by distinct cognitive mechanisms (Cant et al., 2015).

The results of Experiment 3 demonstrated that action-driven attention towards a task irrelevant feature (i.e., color) did not affect the processing of average size. There is, however, some contradictory evidence that suggests irrelevant features can bias ensemble processing. Specifically, Williams et al. (2021) found that holding a color value in visual working memory affects the processing of average orientation. At the start of a trial, participants were shown an irregular shape and asked to study its color and form. Then they were shown a display of 12 bars, consisting of two subsets rotated clockwise or counterclockwise from the global average orientation. The color of one of the subsets of the bars matched the color of the irregular shape held in visual working memory. Participants were then asked to report the average orientation of the entire display. Finally, at the end of the trial, they were shown a new object and asked to report whether it was the same as the object at the start of the trial. Despite the irrelevant relationship between color and orientation, Williams et al. (2021) found that actively maintaining the object in visual working memory influenced the reports of average orientation towards the subset that matched the color of the object held in memory. That experiment showed that an irrelevant feature can influence average orientation reports; however, it is important to note that color was still relevant to the secondary task (i.e., same/different object task) which required participants to hold the color information in visual working memory. In contrast, participants were not required to remember the task-irrelevant color cue in the current study, and thus they would not be motivated to hold any color information in visual working memory during the ensemble task. Thus, the results of Williams et al. (2021) are consistent with the findings of Experiments 1 and 3 of the current study, which explored attentional biases of task relevant and irrelevant cues, respectively. Taken together, we therefore conclude that action-driven attention influences ensemble processing when it is directed toward a task-relevant feature.

Although it was not our main question in Experiment 3, the idea that cueing task-irrelevant features could bias average size reports relates to the impact of selective attention in ensemble processing. Within the literature, there is growing evidence that individual items in an ensemble contribute to the computed average differently, and a possible mechanism for this is selective attention. Notably, Iakovlev and Utochkin (2021) investigated whether salient items in an ensemble would bias average reports. In their study, participants were instructed to report the average orientation of a set of triangles. Importantly, the triangles varied in size, such that the larger items would be amplified by attention, and both the large and small triangles were intermixed in the display. The results demonstrated that the larger items did bias the average reports, however, they also suggested that participants were unable to ignore the less salient items and thus sampled from the entire display. In line with this work, Pasecucci et al. (2021) showed participants an ensemble with two subsets and cued participants to either the internal or the external subset of the ensemble. The two subsets were spatially defined, where the internal subset is closest to the center of the display and the external subset is furthest from the center of the display. When asked to report the average size of the ensemble, participants’ average reports were biased to the cued subset, suggesting they were able to ignore the irrelevant subset. Because the two subsets were visually separate, the authors suggest that when relevant and irrelevant stimuli are not intermixed, ensemble processing can be highly selective and exclusive (Pascucci et al., 2021). Based on the results of these studies, we speculate that the irrelevant feature of color did not bias reports of average size in Experiment 3 because of the intermixing of colored subsets in the ensemble display.

The idea of task relevance is also consistent with the literature surrounding the action effect. Specifically, the biased-competition hypothesis of the action effect (Huffman & Pratt, 2017) seems consistent with the results of Experiment 1 in that the acted-on feature is prioritized by the attentional system when it is behaviorally relevant, compared to behaviorally irrelevant features. Thus a benefit is shown when an action is made towards a congruent cue compared to passively viewing a congruent cue. However, the results of Experiments 2 and 3 are more difficult to explain in this context. Robinson, Clevenger, and Irwin (2018) present an alternative account: the attentional template matching account, in which the goals of the task, rather than the action itself, prioritizes relevant stimuli. In this account, the authors suggest that once participants receive the instructions defining the experimental task, they are used as guidelines to define task-relevant stimuli and the responses made to them within the context of the task instructions (Robinson et al., 2018). Thus, in Experiment 3, when the cued feature is task-irrelevant, it is possible that it does not meet the guidelines set out in the attentional template, and attention does not prioritize these task-irrelevant features. In contrast, the cued feature in Experiment 2 is task-relevant but, the interaction between task and distractor congruency was not significant. While the results showed a trend for lower accuracy for single-size reports when making an action compared with passive viewing in the congruent distractor condition, the critical two-way interaction was not significant. It is possible that this finding is a result of the distinct cognitive mechanisms that govern ensemble processing and single-item processing, however, future research should examine the effect of task-relevant cues on single-item processing (within the broader context of ensemble perception) in greater detail.

In the current study, we use action to direct attention toward a feature that is either relevant or irrelevant to the ensemble phase of the trial. That is, participants either made an action towards a rectangle, allowing attention to be directed toward the size of the shape, or suppressed the action and simply viewed the rectangle on the screen, thus limiting the allocation of attention. While we contend that this attentional manipulation can conceivably affect both the formation of an ensemble representation and subsequent memory-based judgments of it (i.e., reporting average size), one limitation of our paradigm is the inability of our action-based attentional manipulation to cleanly dissociate whether our results are better explained by cognitive mechanisms at perceptual encoding or later decision making (or both equally). Despite this limitation, our results do suggest that attention, while not being necessary for ensemble perception (see Chong & Treisman, 2005; de Fockhert & Marchant, 2008), can indeed affect the pipeline of cognitive processing that leads to the formation, maintenance, and reporting of features from ensemble representations. It will be the task of future research to explore the granularity of this effect, to better localize which cognitive operations are more susceptible to the effects of attention.

The current study makes the case for action-driven attention modulating ensemble processing only when attention prioritizes task-relevant features. Now that we have provided evidence for the involvement of attention in ensemble processing, there is still the question of the mode of attentional deployment that is used during ensemble processing. While most researchers have focused on distributed versus focal attention, there is the additional possibility that object-based or feature-based attention plays distinct (or even interacting) roles in ensemble processing. Future studies should vary the type of attention directed at the same ensemble displays in the same experiment to investigate this further. Finally, it has been established that ensemble processing and scene processing are mediated by shared cognitive and neural mechanisms (Cant & Xu, 2012). Given this functional relationship, it is expected that the action effect will also influence attention in more complex, real-world scenes.

## Conclusions

This set of three experiments found evidence for the involvement of attention in ensemble processing under certain conditions. Specifically, we found that action-driven attention influenced the extraction of summary statistics when directed toward task-relevant cues that elicited an action. Importantly, action-driven attention directed at task-irrelevant cues does not bias reports of average size. Additionally, we found distinct mechanisms involved in processing ensemble and single-item features, such that action-driven attention did not facilitate the processing and recall of the size of single items, but it did for reports of the average size of ensembles. Together, these results provide further insight into the nuanced role attention plays in ensemble processing and, ultimately, advances our understanding of the relationship between visuomotor and perceptual processes.
